# Exploring the impact of early-stage dementia on everyday activities

**DOI:** 10.1177/03080226241261178

**Published:** 2024-07-24

**Authors:** Bethan M Edwards, Monica Busse, Teena J Clouston, Ben Hannigan

**Affiliations:** 1 Service User Research Enterprise (SURE), The Institute of Psychiatry, Psychology and Neuroscience, King’s College London, UK; 2 School of Healthcare Sciences, Cardiff University, Cardiff, UK; 3 Research and Development Department, Cwm Taf Morgannwg University Health Board, Llantrisant, UK; 4 Centre for Trials Research, Cardiff University, Cardiff, UK

**Keywords:** activities of daily living, independence, dementia, occupational therapy, rehabilitation, everyday functioning

## Abstract

**Introduction:**

This paper explores the impact that early-stage dementia has on everyday activities from the perspective of people living with dementia, their supporters and occupational therapy practitioners.

**Method:**

People living with dementia and their supporters (*n* = 10), and occupational therapy practitioners (*n* = 21) took part in semi-structured interviews, with transcripts analysed thematically.

**Findings:**

Six primary themes were identified across participants, namely: (1) ‘Everybody seems to be different, [but] they are similar’; (2) An awareness of change: ‘Something’s not quite right’; (3) ‘*Changes*’ and ‘*difficulties*’ associated with complex and unfamiliar activities; (4) Social withdrawal and exclusion: ‘I’ve felt like I was a leper’; (5) Post-diagnostic mental health: ‘. . .*a dark place*’; and (6) A process of adaptation: ‘I’m still who I am, I can still do things. . .’

**Conclusion:**

Findings indicate that occupational therapy intervention programmes for people living with early-stage dementia should target difficulties associated with a broad range of activity types, and include components that target mental health and motivational needs. The study adds to existing knowledge about the need to personally tailor interventions to ensure that they meet individual needs, experiences, and circumstances. Findings will inform the development of an occupational therapy intervention programme theory (theory of change) for early-stage dementia.

## Introduction

Deterioration in ability to perform everyday activities which significantly impacts independence is a key diagnostic criterion for dementia (World Health Organization, 2021). The International Classification of Functioning, Disability and Health (ICF), defines activity as ‘*the execution of a task or action*’, and broadly proposes activity subcategories which are reflective of the diverse nature of everyday activities, including (but not limited to) mobility, self-care, interpersonal relationships, work, domestic tasks and leisure (World Health Organization, 2024). The phrase everyday activities have been used in this paper to refer to the ICF’s broadly defined concept of activity, whilst recognising that preferences surrounding terminology variers within, and external to, the profession of occupational therapy (e.g., preferences for the terms activities of daily living or occupations).

Impact on everyday activities associated with dementia varies in accordance with stage, dementia sub-type, context, and person; however, people living with early-stage dementia are likely to experience changes in ability performing complex activities for example, community access, managing medication, using technology, and employment for people of working age (Chaplin and Davidson, 2016; Roehr et al., 2019; Silvaggi et al., 2020). Early-stage dementia has also been associated with difficulties performing self-care activities for instance dressing and bathing, albeit less frequently (Giebel et al., 2014). Immediate impacts associated with difficulties performing everyday activities include increased risk, mistakes, non-completion of an activity, feelings of stress and anxiety, as well as the need for support from others (Edwards, 2022). Longer-term consequences include lower quality of life for both caregiver and person living with dementia (Giebel and Sutcliffe, 2018; Giebel et al., 2014), increased apathy and depression (Saari et al., 2020), an increase in caregiver burden (Allen et al., 2019; Lin et al., 2019), and increased paid and unpaid care costs (Michalowsky et al., 2017).

Given the impact early-stage dementia has on everyday activities, being able to continue to engage in everyday activities and do things that they want to do and enjoy, is a priority for people living with dementia in the United Kingdom (UK) (Dementia Diaries, 2024; Reilly et al., 2020). This is consistently emphasised in UK policy and best practice guidelines, for example the Dementia Action Plan for Wales (Welsh Government, 2018), and the Memory Services National Accreditation Programme (MSNAP) (Royal College of Psychiatrists, 2018). In the absence of a cure, and given the adverse impacts associated with everyday activity difficulties, it is vitally important that health and care professionals consider ways in which their interventions meet people living with early-stage dementia’s everyday activity needs.

Occupational therapy is one such intervention which the National Institute for Health and Care Excellence (2018) advises practitioners to consider offering people living with early-stage dementia to support independence and everyday activity functioning. MSNAP Standards for Memory Services also considers input from psychologists and occupational therapists as necessary for the provision of evidence-based psychosocial interventions (Royal College of Psychiatrists, 2018). Despite the emphasis on UK policy and guidance, there is a scarcity of research conducted in a UK context attesting to the efficacy and effectiveness of occupational therapy interventions for this population (Edwards, 2022; Wenborn et al., 2021).

Internationally, existing occupational therapy interventions for early-stage dementia are heterogeneous in nature with variability in mode, duration, intensity, location of delivery and components (Edwards, 2022). Inconsistent outcomes have been reported in differing contexts: for example, Community Occupational Therapy in Dementia (COTiD) was initially evaluated in the Netherlands using a single-site RCT and reported large effect sizes on all outcomes compared to treatment as usual (TAU Graff et al., 2006); however, when evaluated in Germany and the UK using multi-site RCT designs it was no more effective than a 1-hour community occupational therapy consultation in the former (Voigt-Radloff et al., 2011) and TAU in the latter (Wenborn et al., 2021). There is also a tendency for intervention programmes to be designed for people of all stages (Gitlin et al., 2010) or both moderate and early stages (Graff et al., 2006; Voigt-Radloff et al., 2011; Wenborn et al., 2021) as opposed to specific subgroups, for example, early-stage dementia, despite differing everyday activity needs. Reflecting these uncertainties, Sikkes et al. (2021) identified that *‘. . .determining the optimal characteristics of the treatment dose, methods of service delivery, and subgroups most likely to benefit from treatment*’ (p. 264) are key challenges for future research investigating occupational therapy interventions for people affected by dementia.

To address both a gap in the UK evidence base, and international uncertainty about intervention design, outcomes, population, and the impact of context, this study formed part of a larger piece of work to develop an occupational therapy intervention programme theory for people living with early-stage dementia. A programme theory or theory of change ‘. . .*describes how an intervention is expected to lead to its effects and under what conditions*’ (O’Cathain et al., 2019: xxiv), and its identification or development is recommended as an essential activity before an evaluation of complex interventions (Skivington et al., 2021). Programme theories have been notably absent from existing occupational therapy intervention research in this population, necessitating the need to articulate and develop one (Edwards, 2022). This paper reports on preliminary work to this end, consisting of the analysis of data generated during semi-structured interviews about the impact early-stage dementia has on everyday activities.

## Aim

In the context of a larger piece of work which aimed to develop an occupational therapy intervention programme theory for people living with early-stage dementia, this study sought to explore the impact of early-stage dementia on everyday activities from the perspective of people affected by dementia^1^ and occupational therapy practitioners.

## Methods

### Semi-structured interviews

Semi-structured interviews were utilised to generate a detailed understanding of the impact early-stage dementia may have on everyday activity performance (Clark et al., 2021). Interviews have been identified as an accessible way to involve people affected by dementia, particularly given their flexibility to accommodate difficulties concentrating or comprehending (Cridland et al., 2016; Quinn, 2017). Interview schedules were developed with input from the study’s Lived Experience Advisory Group (LEAG) and included questions which were categorised thematically to explore: (1) The impact early-stage dementia has on everyday activities; (2) Interventions delivered by occupational therapists working with people living with early-stage dementia; and (3) The service or practice context, including implementation barriers.

This paper reports on data generated about the impact early-stage dementia has on everyday activities (thematic category 1). Primary interview questions pertaining to this thematic category for occupational therapy practitioners consisted of: ‘*We often hear people talking about early-stage dementia, what does this term mean to you?*’ and ‘*What occupational (activity/functional) needs do you think people living with early-stage dementia have?*’. For people affected by dementia, the words ‘do’ and ‘doing’ were used during interviews rather than ‘occupation’ or ‘activity’, as prior experience interviewing people affected by dementia indicated that these words are often interpreted differently by people who are not occupational therapists. Key questions consisted of the following: ‘*Can you tell me a bit about your day-to-day life, for example what you do in a typical day*?’; ‘*Are there things that you have stopped doing*?’ and ‘*Is there anything you’d like more support doing?’.* Probes were utilised to clarify meanings and expand on pertinent details.

#### Sampling and recruitment strategy

A purposive sampling strategy was used to recruit occupational therapy practitioners from five NHS Health Boards between March 2018 and November 2018 in Wales, UK. Occupational therapy practitioners (occupational therapists and occupational therapy assistants or technicians) working in specialist dementia services were invited to participate by invitation email by the first author (BE) in one Health Board, and in the remaining four, by gatekeepers who were occupational therapy managers. Inclusion criteria were: (1) Working as an occupational therapy practitioner in dementia specialist services; and (2) Willing to participate and able to provide informed consent.

Purposive and snowball sampling strategies were utilised to invite people affected by dementia to participate between November 2018 and January 2020. Inclusion criteria comprised: (1) Has a diagnosis of dementia (any sub-type) OR is a caregiver, family member or a supporter of someone who has; (2) Comfortable talking about their experiences of living with dementia or supporting someone who is; (3) Able to understand written or verbal information in English; and (4) Willing to participate and able to provide informed consent. Recruitment occurred initially through the study’s LEAG who distributed invitations to participate, and by advertising on social media. Given the limited response received from these recruitment methods (*n* = 4), recruitment was initiated through the Join Dementia Research (JDR) database (Join Dementia Research, 2021), however was halted prematurely due to the COVID-19 pandemic. Participants recruited through JDR were approached to participate based upon their identification within the database as someone who is living with early-stage dementia, or a caregiver, supporter or family member of someone who is. JDR is a UK based database that people affected by dementia can use to register their interest in hearing about research that they may be eligible to participate in. Researchers, following ethical approval, can search for potential participants within an agreed distance (7 miles for this study) from their primary recruitment site based on study inclusion/exclusion criteria.

##### Personnel and procedures

Interviews occurred on one occasion on an individual (*n* = 14) and dyad (*n* = 7) basis in accordance with participant preference, with one interview conducted with three participants. Interview location was determined by participant preference and occurred on NHS premises or in the person’s home. Interviews were audio recorded and conducted by the first author (BE) who had an established relationship with occupational therapy practitioners at one participating site as well as three participants affected by dementia through prior engagements with community organisations.

### Data processing and analysis

Miles et al.’s (2014) methods sourcebook for qualitative data analysis was utilised to inform an inductive thematic analysis. Taking a pragmatic approach, they purport that data analysis techniques be used on an *‘as needed basis*’ (p. 9), in a systematic and transparent way. This approach was adopted during this study and described below in four key stages.

#### Stage 1: Processing and preparing

Audio recordings were transcribed by an external contractor specialising in the transcription of health-related interviews (The Transcription Agency, 2022). Anonymised transcripts were uploaded to NVivo to manage and store data.

#### Stage 2: Familiarising and categorising

To enable the analysis of data in accordance with the three thematic categories used to develop research questions (described above), the entire data set was categorised by the first author (BE), and where relevant data could appear in multiple categories. Data was read and re-read and amendments made to data categorisation as necessary. Data pertaining to category 1, the impact early-stage dementia has on everyday activities is reported in this paper, with further findings to be reported elsewhere. During categorisation, provisional coding memos were made in preparation for stage 3.

#### Stage 3: Coding and sub-coding

Data was coded by the first author (BE) using an inductive approach to ensure codes were ‘*grounded empirically*’ in the data (Miles et al., 2014: p81). Data was read and re-read, with provisional codes developed during stage 2 revised iteratively. Final codes and sub-codes with accompanying data extracts were read by authors (BH, TC and MB) who provided feedback on the credibility of codes in light of the raw data, with refinements made as necessary.

#### Stage 4: Narrative description

A narrative description of codes, described as themes is provided in this paper, with differences in views between Health Boards and between participants highlighted. Direct quotations have been reported to provide the reader with an example of the data upon which themes are derived.

### Ethical approval

The study was conducted in accordance with the principles of the Declaration of Helsinki (World Medical Association, 2022) and ethical approval was received from a Health Research Authority (HRA) Research Ethics Committee (REC): REC Reference: 18/WA/0107. Organisational approval was received from all five participating Health Boards’ Research and Development Departments. All participants provided written informed consent to participate in this study.

### Rigour

To enhance trustworthiness, triangulation of data from multiple sources (31 participants), perspectives (practitioners and people affected by dementia) and contexts (five Health Boards) was utilised as a strategy (Lewis et al., 2014). Data was analysed by (BE), who had not worked clinically with this population, thereby minimising bias based on preconceived ideas and opinions. Reflexivity on personal, interpersonal, methodological, and contextual biases and challenges was facilitated throughout the research process through monthly supervision with authors (BH, TC and MB) (Olmos-Vega et al., 2023). Direct quotations have been reported, enabling others to determine if data analysis is supported by the raw data and contradictory perspectives have been highlighted.

## Findings

Twenty-one occupational therapy practitioners participated in this study (Table 1). Nineteen were occupational therapists and two were occupational therapy assistants. Only one participant was male, all were white British or European, and 60% were working as a specialist occupational therapist (NHS band 6) with seniority ranging from assistant (NHS band 3) to highly specialist occupational therapist (NHS band 7). Seven participants reported working in Memory Services (MS); however, only two participants identified MS as their primary practice setting. Mean interview duration was 56 minutes 22 seconds, with a range of 32:44–78:27.

**Table 1.** Demographic data: Occupational therapy practitioners.

Gender	Age	Ethnicity	Highest educational qualification	NHS band	Primary practice setting
Female: *n* = 20 Male: *n* = 1	25–29: *n* = 4 30–34: *n* = 3 35–39: *n* = 2 40–44: *n* = 5 45–49: *n* = 2 50–54: *n* = 3 55–59: *n* = 2	White British / European: *n* = 21	Undergraduate Degree / Diploma: *n* = 18 Post Grad Cert / Diploma: *n* = 3	3: *n* = 2 5: *n* = 1 6: *n* = 12 7: *n* = 6	MS: *n* = 2 Mental Health Liaison: *n* = 3 OP-CMHT: *n* = 7 Inpatient OPMH: *n* = 4 Admissions Prevention: *n* = 2 Young-Onset Team: *n* = 1 Reablement: *n* = 1 Specialist Intervention Team: *n* = 1

MS: memory service; OP-CMHT: older persons community mental health team; OPMH: older persons mental health.

Ten participants affected by dementia participated in the study, five living with dementia and five identified as family members or supporters (Table 2). Three participants living with dementia had a diagnosis of young-onset Alzheimer’s disease (AD) and two with late-onset AD. Two supporters had experience supporting a family member living with Lewy Body Dementia, whilst the remaining three had experience supporting a family member with AD. All participants were white British, and only one male participated. Mean interview duration was 56 minutes 12 seconds, with a range of 29:34–78:09.

**Table 2.** Demographic data: People affected by dementia.

Participants	Gender	Age	Ethnicity	Diagnosis	Time since diagnosis	Relationship with person living with dementia
Person living with dementia	Female: *n* = 4 Male: *n* = 1	50–54: *n* = 2 60–64: *n* = 1 75–79: *n* = 1 80–84: *n* = 1	White British / European: *n* = 5	AD: *n* = 5	Less than 1 year: *n* = 1 1–5 years: *n* = 4	
Caregiver	Female: *n* = 5	18–24: *n* = 1 40–44: *n* = 1 45–49: *n* = 1 70–74: *n* = 1 80–84: *n* = 1	White British / European: *n* = 5	AD: *n* = 3 Lewy Body Dementia: *n* = 2		Child: *n* = 3 Spouse: *n* = 2

AD: Alzheimer’s Disease.

Six themes were identified across participants concerning the impact early-stage dementia has on everyday activities (Table 3), which will be described. Table 3 additionally provides an account of how data from participant groups contributed to the identification and development of themes.

**Table 3.** Early-stage dementia’s impact on everyday activities: Themes identified and nature of contribution per participant group.

Themes	Interviews with people affected by dementia	Interviews with occupational therapy practitioners
	Nature of contribution to theme*	Nature of contribution to theme*
1. ‘*Everybody seems to be different, [but] they are similar’*.	Minor	Major
2. An awareness of change: ‘*Something’s not quite right’*	Major	Major
3. ‘*Changes*’ and ‘*difficulties*’ associated with complex and unfamiliar activities.	Major	Major
4. Social withdrawal and exclusion: ‘*I’ve felt like I was a leper*.’	Major	Major
5. Post-diagnostic mental health: ‘*. . .a dark place*’	Major	Major
6. A process of adaptation: ‘*I’m still who I am, I can still do things. . .*’	Major	Minor

* Major = data made a substantial contribution to theme development. Minor = theme identified in data but did not make a substantial contribution to theme development.

### Theme 1: ‘Everybody seems to be different, [but] they are similar’

A recurring theme across occupational therapy practitioners’ interviews comprised the very individual yet also similar way in which early-stage dementia is experienced and impacts everyday activities. Multiple reasons were provided to underpin this view, including similarities in the way different forms of dementia can impact a person’s skills and abilities during the early stages. For example, occupational therapy practitioners advocated that people living with Alzheimer’s disease (AD) may experience changes to their short-term memory and are therefore likely to experience difficulties remembering medication and attending appointments or events. Conversely, depression, apathy, and difficulties with executive functioning were associated with vascular dementia, whilst participants affected by young-onset dementia and Lewy body dementia described how their mobility and visual perception had been affected:

. . .I can sit on the couch and I can look at the floor, . . . and it looks like it’s just going down, and down, and down, and down . . . I feel like it’s a hole then and I feel like I’m going to fall and I can stand up to go to walk and I will stumble. . .’ (P25, Person living with dementia).

References were made to aspects of a person’s life that can influence impact on everyday activities, including age, education, living and relationship status, level of functioning prior to dementia, and personal interests:

I think it would depend on the person and the individual. I think it would also depend on what their background is to a large extent, but generally if people are still of working age, they’re likely to have difficulties with working and I think somebody with an early dementia perhaps who’s retired is likely to have problems with forgetting possibly appointments. (P05, Occupational therapist).

### Theme 2: An awareness of change: ‘Something’s not quite right’

When discussing very early impacts on activities, including before a diagnosis, lived experiences of a growing awareness that something has changed or that something is ‘*not quite right’* were described. Emphasis was placed on people themselves, or their support network, noticing changes in their ability to complete activities from a previous norm or standard:

. . .people are starting to recognise there’s a problem and they’re starting to look, question what that is . . . what that’s about, looking at information, services, confirming whether it is or isn’t a dementia or something else. . . .it’s the start of signs of changes to day-to-day living. (P11, Occupational Therapist).

Some participants living with dementia described becoming aware that they shared similar symptoms or difficulties with people they knew who had dementia, including family members and friends. Awareness of change was sometimes associated with attempts by people living with dementia to understand and make sense of changes:

It’s a bit of a shock, trying to get your head around it, trying to understand. . . Why you’re not doing the same things that you used to do, why things feel strange, by that I mean the things that you used to do every day, and places you used to go every day, they don’t seem familiar. Trying to understand the mood swings although I don’t realise I’m doing them and everybody else is telling me that I am. (P25, Person living with dementia).

Occupational therapy practitioners also highlighted that some people affected by dementia may not notice that changes have happened themselves. They also emphasised that the person themselves or their family may not be ready to acknowledge that they are experiencing difficulties or feel that any changes are significant enough to seek support or advice during the early stages.

### Theme 3: ‘Changes’ and ‘difficulties’ associated with complex and unfamiliar activities

Frequent references were made to ‘*changes*’, ‘*problems*’ or ‘*difficulties’* in the context of impact on everyday activities. Specifically, ‘c*omplex*’ and unfamiliar or non-routine activities and their environments, were identified by occupational therapy practitioners as potentially difficult for people living with early-stage dementia. Whilst practitioners did not elaborate frequently on what they deemed to be a ‘*complex’* activity, it appeared that these activities relied significantly on higher-order executive functions, with examples provided consisting of work (for people still in employment), domestic activities (e.g. managing finances and medication) activities that involve the use of technology (e.g. cooking), and accessing the community, including driving. Difficulties managing medication were described most frequently across participants:

. . .problems understanding the complexity of the medication, forgetting to take the medication and then if, if something new is introduced, they are struggling. (P06, Occupational therapist).

Examples of unfamiliar or non-routine activities included holidays and appointments, as well as previously familiar activities that had changed, for example washing in a shower instead of a bath. Activities that were previously carried out by others, and therefore unfamiliar, could also be challenging:

P30: “. . .my Dad always did the bills again, so anything to do with money, anything new and she gets confused.”

P31: “The television I get confused with.”

P30: “Because again Dad did the recordings and, yeah. . .Another thing is central heating timer. Now, if it needs to be changed, like when the clocks change. . .” (P30, Family member and P31, Person living with dementia).

Orientation in an unfamiliar environment, for example when driving or when out in the community was a commonly reported experience. Nevertheless, whilst some participants affected by dementia reported difficulties carrying out new and unfamiliar activities, others highlighted that with the right social support, these are activities that they can continue to perform and enjoy:

. . .our friends . . . we go on holidays with them and they understand that. . . if I want to go bed in the day, I can go, they, I got no pressure, and they watch over me as well. So I’m lucky, there’s some people haven’t got anyone . . . (P22, Person living with dementia).

‘Changes’ and ‘difficulties’ during activity performance were associated with making mistakes, the increased involvement of others as sources of support and not initiating or completing activities. Sometimes the person’s safety or wellbeing could be compromised for example getting lost when out in the community or through omission or repetition:

. . .I forget my tablets, I’ll take my tablets and then I think, have I taken my tablets, and then I’ll take them again, so I overdose myself. (P25, Person living with dementia).

### Theme 4: Social withdrawal and exclusion: ‘I’ve felt like I was a leper.’

The cessation of, or the reduction in, social and leisure activities was frequently described in the context of early-stage dementia. Occupational therapy practitioners spoke about the impact early-stage dementia can have on ability to communicate, for example forgetting names and words, which could lead to social withdrawal:

. . .Maybe they’re not keeping up with the conversation or they’re feeling a bit embarrassed because they’re having trouble remembering people’s names or topics or dates of groups and things. So we’d expect maybe a little bit of withdrawal from social activities as well. (P06, Occupational therapist).

Participants living with dementia spoke about the impact early-stage dementia had had on their relationships with family and friends including how roles and responsibilities within a family had changed, for example a daughter providing care for a parent. A decreasing social circle was described after diagnosis by participants living with young-onset dementia: ‘*All my work colleagues gone . . . I’ve felt like I was a leper*.’ (P25, Person living with dementia). Two participants with young-onset dementia spoke about being excluded from their work environments and the lack of support and adaptations available to them to remain in employment:

. . .they forced me into early retirement which I didn’t want to do and in reality now, in hindsight they should have . . . put me in a different position and kept me earning, and all they wanted to do was get rid of me because I was dead wood. . . (P23, Person living with dementia).

### Theme 5: Post-diagnostic mental health: ‘. . .a dark place’

Whilst depression, anxiety, and apathy can be experienced as a symptom of early-stage dementia, participants affected by young-onset dementia also spoke about ‘*feeling emotional*’ and going into *‘. . .a dark place’* after being diagnosed with a degenerative condition at a young age. Poor mental health was also described by these participants as a consequence of the loss of previously held roles and activities, for example work and hobbies. Impact on everyday activities associated with poor mental health (as a primary symptom of dementia and as a consequence of diagnosis and increased disability) was associated with low motivation which in turn impacted on ability to initiate, carry out and complete a range of activities:

I didn’t get out of bed, I wouldn’t get out of bed, I didn’t do any cooking, cleaning, I wouldn’t get out of bed. (P23, Person living with dementia).

Some participants affected by dementia described experiencing greater conflict in their relationships with family, which they attributed to poor mental health and a lack of information and support for families. Others spoke about the cumulative impact that their declining functional ability had upon how they viewed themselves and the impact they were having on others. One participant spoke about her concerns of becoming a ‘burden’ to her family:

I can see me going downhill. I can’t afford to, not yet. I know it’s going to happen, and I know, this is the only thing that cripples me is that I’m going to be a burden to the family. (P22, Person living with dementia).

Nevertheless, not all participants reported experiencing poor mental health, with receiving support immediately after diagnosis as well as older age at onset, appearing to influence this impact. One participant recalled: ‘*I took it on the chin right, but then I did get all the correct help that I needed*’ (P22), whilst another stated *‘. . .I more or less accepted . . . I was upset, but I was expecting it . . . I think because of the family history as well’* (P31).

### Theme 6: A process of adaptation: ‘I’m still who I am, I can still do things. . .’

Whilst participants living with dementia reported experiencing changes in their ability to carry out their everyday activities, they also expressed a determination to remain active, with a process of adaptation evident since diagnosis. For example, participants living with young-onset dementia spoke about new roles and activities, including attending and facilitating peer support groups:

. . .you get to know them [peer support group members], and we understand one another. I’ve . . . learnt one of them to crochet. She crocheted slippers . . .and she’s crocheted a blanket for her mother, shawl. But that’s something that’s in me, I like to help others, and I suppose that’s not going to go away easily. . . (P22, Person living with dementia).

Whilst peer support was an activity through which one participant could maintain their identity as someone who helped others, another participant living with young-onset dementia spoke about experiencing a change in their career and new identity as a patient advocate:

I haven’t got time to work anyway because I’m still busy so it’s like . . .my career path now is, give my name, give my word and speak. . . (P23, Person living with dementia).

Further examples of adaptation were described by participants affected by young and older-onset dementia, consisting of self and supporter-initiated adaptations to the way everyday activities were performed. Participants described using calendars, dosette boxes and electronic reminders to support everyday activity performance. One participant described how he continued to engage in his two life-long interests: woodwork, and walking, however, required verbal support from family to complete these activities:

. . .we discuss it more which we never used to do, yeah, because he would get on with it and do it, where now. . . (P29, Family member).

Whilst self and caregiver-initiated adaptations were described by some occupational therapy practitioners, they noted that these adaptations did not always support everyday activity performance:

. . .you might find that they have started [using a diary] but they’ll lose it or they won’t start using it again. And so they might have three different diaries on the go at the same time and different information and different things [in each]. (P10, Occupational therapist).

## Discussion

This qualitative study, consisting of semi-structured interviews with occupational therapy practitioners and people affected by dementia aimed to explore the impact early-stage dementia has on everyday activities. Six primary themes were identified which will be discussed in relation to existing research and the generation of new knowledge. These interviews were conducted in the context of a larger piece of work which aimed to develop an occupational therapy intervention programme theory (theory of change) for people living with early-stage dementia and therefore implications for this work will also be considered.

Existing qualitative research in this area has been conducted primarily in Sweden, using observational methodologies as well as interviews with people affected by dementia (Brorsson et al., 2011, 2013; Nygård and Johansson, 2001; Nygård and Öhman, 2002; Nygård and Starkhammar, 2003, 2007; Vikström et al., 2008). This study builds on the findings of this body of research in a new context (the UK), as well as by including the perspective of occupational therapy practitioners. This existing body of research has also primarily investigated impact on specific activities, namely accessing the community (Brorsson et al., 2013; Brorsson et al., 2011), and activities involving everyday technology (Nygård, 2008; Nygård and Starkhammar, 2003; Nygård and Starkhammar, 2007; Rosenberg et al., 2009). By taking a broader approach, this study extends knowledge about the nature of the contexts and everyday activities associated with changes, for example, those associated with complexity, experienced as new and unfamiliar, as well as exclusion from social activities and relationships. This study indicates therefore that an occupational therapy intervention programme theory for early-stage dementia should include components that target a broad range of everyday activities and should not be confined to personal care activities and domestic activities (e.g. medication management and meal preparation) as is common in clinical practice in the UK (Edwards, 2017; Swinson et al., 2016). Whilst methods of change targeting activities were not explored in this paper, participants affected by dementia described implementing their own problem-solving strategies consisting of modifying how an activity is performed (e.g. with support from others, prompts and reminders).

Theoretical models of loss and grieving have been used by Robinson et al., (2005) and Yuile (2019) to discuss the process of noticing changes, coming to terms with an eventual dementia diagnosis, and adapting to changes. This study’s second, fifth and sixth themes reflect elements of this process in the context of impact on everyday activities and therefore these models may offer important insight when further developing an occupational therapy intervention programme theory. At the time of interview, it appeared that the most recently diagnosed participant living with dementia (P25) was still coming to terms with their dementia diagnosis, whilst the remaining participants living with dementia appeared to have moved to an adapting stage. This suggests that a programme theory should permit tailoring to where individuals are in the process of noticing changes, coming to terms with a diagnosis, and implementing adaptations. Occupational therapy interventions delivered shortly after diagnosis may therefore need to include components that support movement through this process, addressing any mental health and motivational needs impacting everyday activities, particularly in contexts where existing post-diagnostic support is scarce and when working with people living with young-onset dementia. Again, methods of change were not specifically explored, however, a normalising and identifying with others strategy was used by two participants living with late-onset dementia to make sense of everyday activity changes which they found helpful. Whilst these strategies were also described as helpful for some of Öhman et al. (2008) participants, other participants found identifying with others to be a distressing experience. Indeed, this process may be more profound for people living with young-onset dementia, who are less likely to find normalising strategies helpful, may have dependent children, be in employment, and have experienced greater difficulties in obtaining a diagnosis (Yuile, 2019).

Akin to the principles of person-centred care (Kitwood, 1997) and consistent with best practice guidelines National Institute for Health and Care Excellence (2018), this study’s first theme described views expressed across participant groups about the individual way in which early-stage dementia impacts everyday activities, reflecting the wider literature about factors that influence experiences of dementia, access to services, and the tailoring of interventions (Cooper et al., 2010, 2015; Hasselgren et al., 2018; Hutchinson et al., 2019). This underscores the importance of occupational therapy interventions which incorporate a programme theory that represents and facilitates personalisation and tailoring. In particular, this study has highlighted that adaptations to intervention content and components may be shaped by dementia sub-type, age at onset, where a person is in coming to terms with a diagnosis, post-diagnostic mental health, and the type of everyday activities impacted. These adaptations may impact on the duration and intensity of the delivered intervention – if for example, additional intervention components targeting mental health needs are required.

### Limitations

Recruitment of participants was halted due to the COVID-19 pandemic, resulting in only 10 participants affected by dementia, whilst 21 occupational therapy practitioners participated. Reflecting the ethnicity of the population living in the geographical area where recruitment occurred (which in the 2021 census was over 96% white), participants affected by dementia were all white British or European. Only one male took part and only one participant was living on their own. Caution therefore should be taken when generalising from this study to people affected by dementia who do not share the characteristics of the study population. Indeed, increased everyday activity difficulties have been reported among people living with dementia or cognitive impairment who live alone compared to those living with others (Yang et al., 2022), and among women who are black and Hispanic, when compared to white women (Edwards et al., 2020). Both Yang et al. (2022) and Edwards et al. (2020) suggest that these disparities are a consequence of unmet everyday activity needs due to inequalities in the availability of, and access to, appropriate health and social care services for these populations. Given the dearth of qualitative studies investigating impact on everyday activities and early-stage dementia, it is imperative that future qualitative studies ensure that the experiences of people living alone and the experiences of the global majority are centred.

Alternative methods of generating data were recommended by the study’s LEAG, for example video diaries, however given the confines of time and resources this was not possible. Future studies may wish to consider using alternative or supplementary methods of generating data in consultation and collaboration with people affected by dementia. It is also important to reiterate that data were analysed in the context of a programme of work seeking to develop an occupational therapy intervention, and secondary analysis of the data may glean new insights into the views and experiences of participants.

## Conclusion

To the authors’ knowledge, this is the first qualitative interview study exploring the impact early-stage dementia has on everyday activities from the perspective of occupational therapy practitioners and people affected by dementia in a UK context. In addition to generating data in a new context and from new perspectives, this study has generated novel insights about the impact poor mental health and motivation secondary to receiving a diagnosis can have on everyday activities, as well as the impact early-stage dementia can have on activities described as complex, unfamiliar, non-routine, and social activities and relationships. These findings suggest that occupational therapy interventions for early-stage dementia targeting everyday activities should not be confined to personal care activities and domestic activities (e.g. medication management and meal preparation), and should also include components to meet mental health needs. This study also adds to existing knowledge about the need to personally tailor intervention programmes to ensure that they meet personal needs, experiences, and circumstances, with findings from this study informing the development of an occupational therapy intervention programme theory for early-stage dementia.

**Key findings**

- Difficulties performing a broad range of everyday activities were associated with early-stage dementia.
- Secondary impacts on everyday activities were reported due to poor mental health following diagnosis and the reaction of others.
- People affected by early-stage dementia implement their own problem-solving strategies of varying success.

**What the study has added**

Insight into the impact early-stage dementia has on everyday activities from the perspective of occupational therapy practitioners, people living with dementia and their supporters in a UK context.

## Notes

1. On the recommendation of the study’s Lived Experience Advisory Group (LEAG), the term ‘people affected by dementia’ in this study refers to people living with dementia as well as people who identify as supporters, family, friends, or unpaid caregivers.
